# Long Terminal Repeat Circular DNA as Markers of Active Viral Replication of Human T Lymphotropic Virus-1 *in Vivo*

**DOI:** 10.3390/v8030080

**Published:** 2016-03-14

**Authors:** James M Fox, Silva Hilburn, Maria-Antonietta Demontis, David W Brighty, Maria Fernanda Rios Grassi, Bernardo Galvão-Castro, Graham P Taylor, Fabiola Martin

**Affiliations:** 1 Centre for Immunology and Infection, Department of Biology & Hull York Medical School, University of York, York YO10 5DD, UK; james.fox@york.ac.uk; 2 Section of Infectious Diseases, Department of Medicine, Imperial College, London W2 1PG, UK; s.hilburn@imperial.ac.uk (S.H.); m.demontis@imperial.ac.uk (M.-A.D.); g.p.taylor@imperial.ac.uk (G.P.T.); 3 The National Centre for Human Retrovirology/HTLV Clinic, Imperial College Healthcare NHS Trust, St Mary’s Hospital, London W2 1NY, UK; 4 Division of Cancer Research, Ninewells Hospital & Medical School, University of Dundee, Dundee DD1 9SY, UK; d.w.brighty@dundee.ac.uk; 5 Oswaldo Cruz Foundation, Gonçalo Moniz Research Center-BA, Advanced Laboratory of Public Health, Salvador, Bahia, Candeal 40296-710, Brazil; grassi@bahia.fiocruz.br; 6 HTLV-Centre Bahian School of Medicine, Salvador, Bahia 40050-420, Brazil; galvao@bahiana.edu.br

**Keywords:** long terminal repeat, LTR DNA circles, human T-lymphotropic virus, HTLV, viral replication

## Abstract

Clonal expansion of human T-lymphotropic virus type-1 (HTLV-1) infected cells *in vivo* is well documented. Unlike human immunodeficiency virus type 1 (HIV-1), HTLV-1 plasma RNA is sparse. The contribution of the “mitotic” spread of HTLV-1 compared with infectious spread of the virus to HTLV-1 viral burden in established infection is uncertain. Since extrachromosomal long terminal repeat (LTR) DNA circles are indicators of viral replication in HIV-1 carriers with undetectable plasma HIV RNA, we hypothesised that HTLV-1 LTR circles could indicate reverse transcriptase (RT) usage and infectious activity. 1LTR and 2LTR DNA circles were measured in HTLV-1 cell lines and peripheral blood mononuclear cells (PBMC) of asymptomatic carriers (ACs) and patients with HTLV-1-associated myelopathy/tropical spastic paraparesis (HAM/TSP) or adult T cell leukaemia/lymphoma (ATLL). 1LTR DNA circles were detected in 14/20 patients at a mean of 1.38/100 PBMC but did not differentiate disease status nor correlate with HTLV-1 DNA copies. 2LTR DNA circles were detected in 30/31 patients and at higher concentrations in patients with HTLV-1-associated diseases, independent of HTLV-1 DNA load. In an incident case the 2LTR DNA circle concentration increased 2.1 fold at the onset of HAM/TSP compared to baseline. Detectable and fluctuating levels of HTLV-1 DNA circles in patients indicate viral RT usage and virus replication. Our results indicate HTLV-1 viral replication capacity is maintained in chronic infection and may be associated with disease onset.

## 1. Introduction

Human T-lymphotropic virus type-1 (HTLV-1) is a complex retrovirus, in the genus *Deltaretrovirus* of the subfamily *Orthoretrovirinae* of the *Retroviridae* family of viruses. It is an oncovirus causing adult T cell leukaemia/lymphoma (ATLL) [1,2] and is associated with a variety of inflammatory conditions, most notably HTLV-1-associated myelopathy/tropical spastic paraparesis (HAM/TSP) [3,4]. Retroviruses carry a diploid plus-strand RNA genome [5] as well as viral enzymes reverse transcriptase (RT), integrase, protease and RNase H. Once released into the cytoplasm, viral RNA is transcribed into double stranded linear DNA flanked directly by long terminal repeats (LTRs). The DNA is transported into the nucleus within the pre-integration complex. In the nucleus the linear un-integrated DNA is either inserted into the host genome or circularised (Figure S1) [6,7,8]. The circular DNA has been found in different configurations such as 1LTR [7,9,10], tandem 2LTR [11,12] and heterogeneous 2LTR DNA circles [13] depending on the number and position of the LTRs. They are generally assumed to be dead-end by-products but it has been suggested that they are functional or even cytotoxic [14,15]; at times, un-integrated DNA can represent 99% of total viral DNA [16].

Human immunodeficiency virus type 1 (HIV-1) 2LTR circles have been used as an experimental marker of RT activity [17], the nuclear transport of linear DNA [18] and the ongoing replication of the HIV-1 latent reservoir in patients on antiretroviral therapy [19]. HIV-1 2LTR circle concentration decreases with RT inhibition and increases with integrase inhibition; its consistent detection and change in concentration shows that even with long-term quadruple antiretroviral therapy HIV-1 continues to produce new DNA and is therefore not latent [19].

Recent reviews model HTLV-1 replication [20,21] but, at least *in vivo*, HTLV-1 viral replication capacity is still not fully understood. HTLV-1 transmission is known to be preferentially cell associated as cell free products of HTLV-1 patients have often, though not always [22], been found to be non-infectious [23]. More recently, however, cell-free virus biofilm assemblies have been shown to be capable of infecting lymphocytes and, by preference, dendritic cells [24]. HTLV-1 RNA is virtually undetectable in patients’ plasma [25] leading to the assumption of no or little RT usage *in vivo*. However, we believe that a number of viral replicative cycles must occur in a new host to establish infection [26] and sequence variations have been observed within families following mother-to-child transmission (MTCT) [27]. Indeed, the kinetics of acute HTLV-1 infection in patients that had received organ transplantation from an HTLV-1 infected individual have recently been demonstrated, revealing that proviral load set point and seroconversion was reached within six weeks through clonal expansion but also via a high rate of infectious spread [28].

The HTLV-1 viral load is reported as the total viral DNA detected in 100 peripheral blood mononuclear cells (% PBMCs) often referred to as proviral load (PVL). This can vary from <0.001% to >100%; is known to be low (<1%) in 50% of asymptomatic carriers (ACs) and high (>1%) in all patients with ATLL and HAM/TSP, but remains remarkably stable over several years in patients without ATLL [29]. Several HIV-1 antiretrovirals have been shown to be effective HTLV-1 enzyme inhibitors *in vitro* [30,31]. However no drug intervention to date has consistently decreased the HTLV-1 total DNA in humans [32,33,34,35], although a significant reduction was detected in STLV-1 infected macaques treated with sodium valproate (used as a histone deacetylase inhibitor) and the nucleoside analogue RT inhibitor, zidovudine [36]. HTLV-1 infection is maintained through Tax driven clonal expansion of HTLV-1 infected CD4+/CD25+ T cells, but the virus is able to spread *in vitro* from cell-to-cell through the virological synapse [37,38,39,40] and through the exchange of transient biofilm-like extracellular virus particles held on the surface of an infected cell [41]. How much this spread is contributing to the DNA concentration *in vivo* remains unknown [42]. An interference with viral replication may reduce transmission and the risk of developing HTLV-1 associated disease through resetting the total DNA burden at a lower set point.

The detection and quantification of 1 and 2LTR HTLV-1 DNA circles *in vivo* could be a biomarker of continuous HTLV-1 viral replication in parallel to its mitotic expansion; this study was designed to address this hypothesis.

## 2. Materials and Methods

### 2.1. Cell Culture

MT-2 cells were cultured at 37 °C with 5% CO_2_ in Roswell Park Memorial Institute (RPMI) 1640 medium, supplemented with 100 IU/mL penicillin, 100 μg/mL streptomycin, 2 mM L-glutamine, 10 mM HEPES and 10% heat inactivated FCS; HuT 102 cells were cultured in the same media with the inclusion of 1 mM sodium pyruvate and 4500 mg/mL glucose (all cells were purchased from the ATCC, Teddington , UK; reagents were from Invitrogen, Paisley, UK or PAA Laboratories, Yeovil, UK).

### 2.2. In-Vivo Studies

Stored DNA samples from PBMCs of 48 HTLV-1 positive, HIV negative, patients were tested retrospectively. Forty-seven samples donated by patients attending the National Centre for Human Retrovirology (NCHR) at Imperial College Healthcare NHS Trust, St Mary’s Hospital, London, UK and DNA from one patient at the HTLV Centre at Bahiana Medical School, Salvador, Brazil were studied. All samples were donated after written informed consent to participate in research approved by the UK National Research Ethics Service, Oxford “A” Local Research Ethics Committee (Ref: 09/H0606/106) or the institutional review board of the Oswaldo Cruz Foundation, and the National Commission on Ethics in Research (CONEP record: 15271), Brazilian Ministry of Health. Insufficient DNA was available to test all samples for 1 and 2LTR. For the 1LTR studies 20 samples were available: six patients were ACs, ten had HAM/TSP and four ATLL; for the 2LTR studies 31 patient samples were available: 13 ACs, ten HAM/TSP and eight ATLL (five with lymphoma, an aggressive type of ATLL and three with more indolent disease: one with chronic ATLL in complete remission and two with cutaneous lymphoma). Four samples were duplicated in 1LTR and 2LTR testing, giving a total of 47 patient samples.

### 2.3. DNA Extraction and Amplification

All DNA were extracted from the cell lines and PBMCs as previously published [43]. Circular, low molecular weight DNA was extracted from total genomic DNA through QIAprep spin miniprep kit (Qiagen, Manchester, UK) according to the manufacturer’s protocol and quantified using a nanodrop spectrophotometer (ThermoScientific, Loughborough, UK). Primers (Table 1 and Figure S1) for detection of 1 and 2LTR HTLV-1 DNA circles using classical and nested PCR were designed using Primer3 [44,45] by alignment of the AKT strain of the complete HTLV-1 genome, accession number J02029.1 [5]. For the detection of 1LTR circle DNA using the classical PCR protocol the primers were designed in the pX and gag region. For the 2LTR DNA circles, primers were seated either side of the LTR-LTR junction. Primers used for the detection of low copy number 1 and 2LTR DNA circle in the nested PCR protocol were designed in the same fashion (Table 1). National Center for Biotechnology Information (NCBI) Blast database program was used to confirm that the designed sequences did not match other published DNA sequences. Primers were manufactured by Invitrogen or Sigma (Dorset, UK). Concentrations of LTR circles were measured at two time points, immediately and four hours post venepuncture, to observe whether there were time dependent variations.

### 2.4. PCR Reaction Protocol

Classical PCR: DNA extracted from 6 × 10^5^ cells was used for amplification in 50 μL reaction volumes containing 100 pM of each primer, 200 μM dNTPs, 2 mM MgCl, 1 x green GoTaq reaction buffer (Promega, Southampton, UK) and 1U GoTaq polymerase (Promega). The cycling conditions on an MJ research PTC 200 Thermal Cycler were: denaturation step 5 min at 94 °C, followed by 35 cycles of amplification consisting of 1 min at 95 °C, 30 s at 62 °C (1LTR) or 66 °C (2LTR), 5 min at 72 °C, and a final elongation step at 72 °C for 5 min. Where necessary, one microliter of the first round (classical) product was transferred to 49 μL of reaction mix containing the reagents at the same final concentrations as described for the classical PCR for the nested PCR. Cycling conditions for the nested PCR were as follows: denaturation step 5 min at 94 °C, followed by 35 cycles of amplification consisting of 1 min at 95 °C, 30 s at 66 °C, 5 min at 72 °C, and a final elongation step at 72 °C for 5 min. 12 µL from each reaction was separated on a 2% agarose gel (NuSieve 3:1, Lonza, Cambridge, UK) and visualised by ethidium bromide (Sigma) staining under UV light using the Syngene gel documentation system and GeneSys software. The identity of the amplicons were confirmed through purification of the PCR product from the gel through PCR Purification Kit (Qiagen) according to the manufacturer’s protocol and sequencing 400 bp on each side with the forward and reverse primers. The sequencing was carried out by the MRC CSC Genomic Core Laboratory at Hammersmith Hospital, London, UK. Standard sequencing generates 700–800 bases of readable sequence from a single sequence. The NCBI Blast program was used to identify the DNA sequence.

### 2.5. Quantification of LTR DNA Circles and HTLV-1 PVL

LTR DNA circles were determined by serial dilution of purified sample DNA in distilled water followed by amplification of quadruplicates at each dilution. The quantity of LTR circles were determined from Poisson’s distribution at the lowest concentration of DNA that resulted in amplification in at least one of the replicates, where load = −log_n_·Fo^x^ dilution, and Fo is the number of negative tests/the number of tests. HTLV-1 PVL was measured using real-time PCR (LightCycler, Roche, Mannheim, Germany) as previously described [46].

### 2.6. Controls

One hundred base-pair DNA was designed to spike HTLV-1 negative DNA as positive controls for primer amplification. MT-2 cells were found to contain LTR DNA circles and were used as positive controls thereafter. Negative controls were DNA isolated from HTLV-1 negative cord and donor PBMCs or water.

### 2.7. LTR DNA Localisation

MT-2 cells were treated with triton-sucrose buffer (0.32 M sucrose, 1% Triton X-100, 5 mM MgCl2, 10 mM Trio-HCL, pH 7.5) and centrifuged for 1 min at 14,000 g in order to separate cytoplasm from nucleus. DNA was extracted using Qiagen DNA mini kit according to the manufacturer’s protocol and 1LTR DNA circles were quantified as above.

### 2.8. Reverse Transcriptase Inhibitory Study

Tenofovir disoproxil fumarate (TDF) was obtained through the AIDS Research and Reference Reagent Program, NIAID, NIH. MT-2 cells were cultured in the presence or absence of TDF at its known inhibitory concentration 50 (IC50), 0.01 µmol/L with media changes and TDF refreshment at three-day intervals. Growth rates were compared by taking regular cell counts. 1LTR DNA circles were quantified in MT-2 DNA extracted and purified by QIAprep spin miniprep on days 0, 17 and 28.

### 2.9. Cell Entry Inhibitor Study

MT-2 cells were cultured as previously described for 10 days with and without 10 μM P^cr^-400 with media changes and drug refreshment at three-day intervals [47]. Aliquots of 6 × 10^6^ cells were obtained on days 3, 7 and 10; DNA was extracted and purified from both treated and control cells by QIAprep spin miniprep and 1LTR DNA circles were quantified as described above.

### 2.10. Statistical Analysis

Data were analysed in SPSS (v14) or GraphPad Prism (v6.02) using parametric (*t*-test) and linear regression model for comparing two or more continuous variable. Non-parametric tests (Mann-Whitney Test) were used for correlating categorical with continuous variables and chi-square (Pearson Chi-Square Test for large >5, and Fisher’s Exact test <5 sample size) for categorical variables. One way non-parametric ANOVA tests with Dunn’s multiple comparison correction or non-parametric *t*-test were used where appropriate. Results were considered statistically significant if a *p*-value < 0.05 was achieved.

## 3. Results

### 3.1. Detection, Localisation, Quantification of LTR DNA Circles

We previously presented preliminary data on circularised LTR DNA concentration in MT2 cells [48] but now with revised methodology and using matched cell extracts we have found both 1 and 2LTR DNA circles in two cell lines: MT-2 and HuT 102 (Table 2) originally derived from HTLV-1 infected individuals [2,49,50]. 1LTR DNA circles were approximately half as frequent as 2LTR DNA circles in MT-2 and HuT 102 cell lines. In localisation studies, LTR DNA circles were detected at 1 copy/6000 MT-2 cells in cytoplasm and at 1 copy/87 MT-2 cells in the nuclei.

### 3.2. Biomarker for Persistent RT Usage

MT-2 cells were cultured with and without TDF, a potent RT inhibitor, or P^cr^-400, a HTLV-1 entry inhibitor at their respective inhibitory concentration 50 (IC50); the drugs did not impair cell proliferation or viability (data not shown). Whilst LTR circle concentration decreased in the presence of TDF: 1 copy/0.6 MT-2 cells (day 0) compared with 1 copy /2.1 MT-2 cells (day 17 and day 28), it did not change over time with entry inhibitor P^cr^-400 indicating, albeit futile, persistent viral RT enzyme usage and potential recycling of virus intracellularly.

### 3.3. LTR DNA Circles in HTLV-1 Infected Patients

In total, PBMCs of 47 chronically infected HTLV-1 carriers were tested. 1LTR DNA circles were measured in 20- and 2LTR DNA circles in 31-PBMC samples with dual testing in four samples. Patient demographics are outlined in Table S1. In order to detect a potential decrease of LTR DNA circle concentration in blood samples over time due to sample aging, LTR DNA circle levels were compared in four blood samples where DNA was extracted immediately or after a four hour delay post venepuncture of patients. LTR DNA circle levels were found to be independent of the timing of DNA extraction (data not shown).

**Figure 1:**
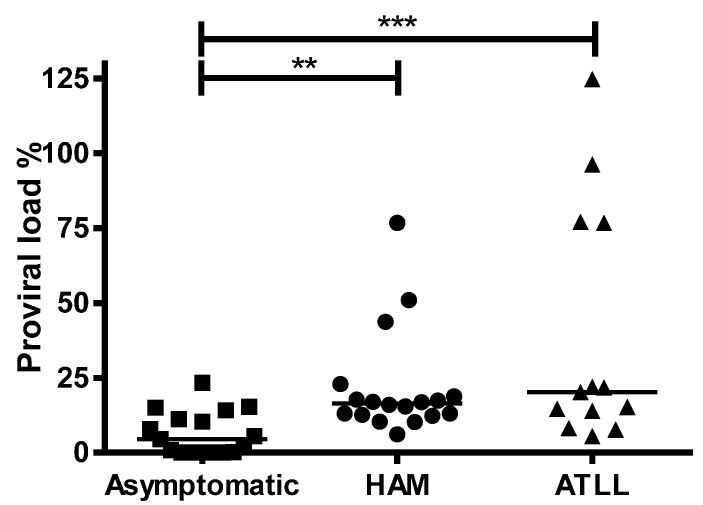
Human T-lymphotropic virus type-1 **(**HTLV-1) proviral load (PVL) is higher in HTLV-1 infected patients with HTLV-1 associated disease than in asymptomatics. PVL from patients in three diagnostic groups: asymptomatic (*n* = 19), HTLV-1-associated myelopathy/tropical spastic paraparesis (HAM/TSP) (*n* = 20) or adult T cell leukaemia/lymphoma (ATLL) (*n* = 12) are displayed. Horizontal line represents median PVL within each group. Statistical differences between groups were calculated using non-parametric one-way ANOVA with Dunn’s multiple comparison correction. **: *p* < 0.01, ***: *p* < 0.001.

As expected [29], PVL was significantly lower, *p* = 0.0019, among ACs (median of 4.5%) compared with patients with HAM/TSP (median of 16.40) or ATLL (median of 20.20), *p* = 0.0010, with no significant difference between HAM/TSP and ATLL patients (Figure 1). 1LTR DNA circles were detected in 14/20 (67%) patients: 4/6 (67%) ACs, 7/10 (70%), HAM/TSP and 3/4 (75%) ATLL. The presence of 1LTR DNA circles did not differ between ACs and patients with HAM/TSP (Fisher’s Exact test, *p* = 0.15) or ATLL (*p* = 0.6). 1LTR DNA circle concentration did not differ significantly between the ACs, HAM/TSP or ATLL groups and was not a predictor of clinical status (Table 3).

**Figure 2:**
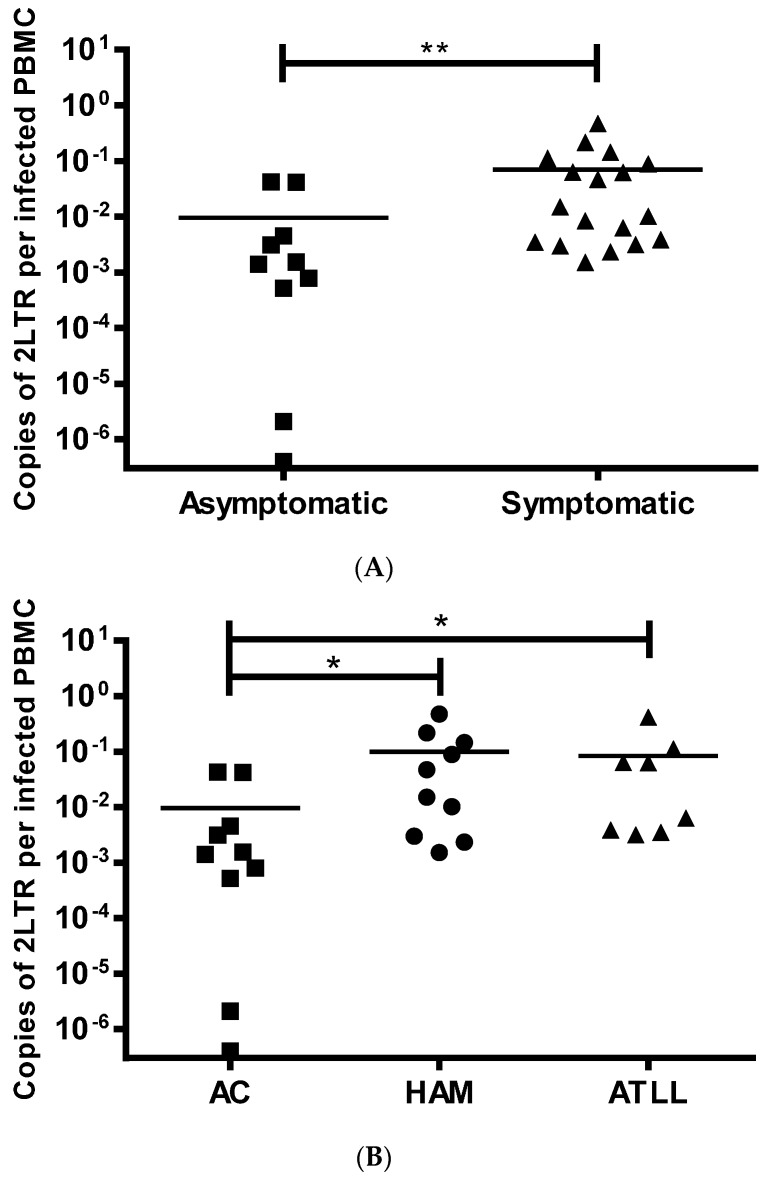
2LTR DNA circle level is higher in HTLV-1 infected patients with HTLV-1 associated diseases compared to asymptomatics. In panel (**A**), 2LTR DNA circle levels in individual patients are plotted for asymptomatics (■ *n* = 10) or symptomatics (▲ *n* = 18). In (**B**), the same data is plotted for patients diagnosed as asymptomatic (■), HAM/TSP (● *n* = 10) or ATLL (▲ *n* = 8). Mean 2LTR DNA circle levels within each group are depicted by the horizontal bar; statistical comparisons are performed using non-parametric *t*-test (**A**) or a non-parametric one-way ANOVA with Dunn’s multiple comparisons correction. *: *p* < 0.05, **: *p* < 0.01.

2LTR DNA circles were detected in 28/31 (90%) PBMCs from patients using classical PCR and in 30/31 (97%) by nested PCR. 2LTR DNA circles were not detected even with nested PCR in one patient, who had a PVL of 0.01%. 2LTR DNA circles were detected at significantly higher concentrations in patients with diagnosed disease (HAM/TSP and ATLL) compared to ACs (*p* = 0.003) (Figure 2A), whilst there was no significant difference between patients with HAM/TSP and ATLL (Figure 2B). This association with disease was also detected when ACs with high PVL, similar to patients with HAM/TSP, were compared to HAM/TSP and ATLL patients (Table 4). It is important to point out that none of the patients with ATLL suffered from the acute leukemic form and that the patient with chronic ATLL was in complete remission. Therefore the DNA was extracted from non-cancerous HTLV-1 infected PBMCs reflecting the background HTLV infection rather than the transformed cells. This makes the results comparable to asymptomatic patients and those with HAM/TSP.

From our patient samples, we calculated that based upon patient PVL, on average, DNA from 529 ± 296 infected cells (2800 ± 1350 total PBMCs) were required to detect one 2LTR DNA circle in ACs. DNA from 163 ± 73 (1811 ± 852 PBMCs) infected cells were required for the detection of one 2LTR DNA circle in patients with HAM/TSP and DNA from 148 ± 49 (1484 ± 477 PBMCs) infected cells were required for detection of one 2LTR DNA circle in patients with ATLL.

**Figure 3:**
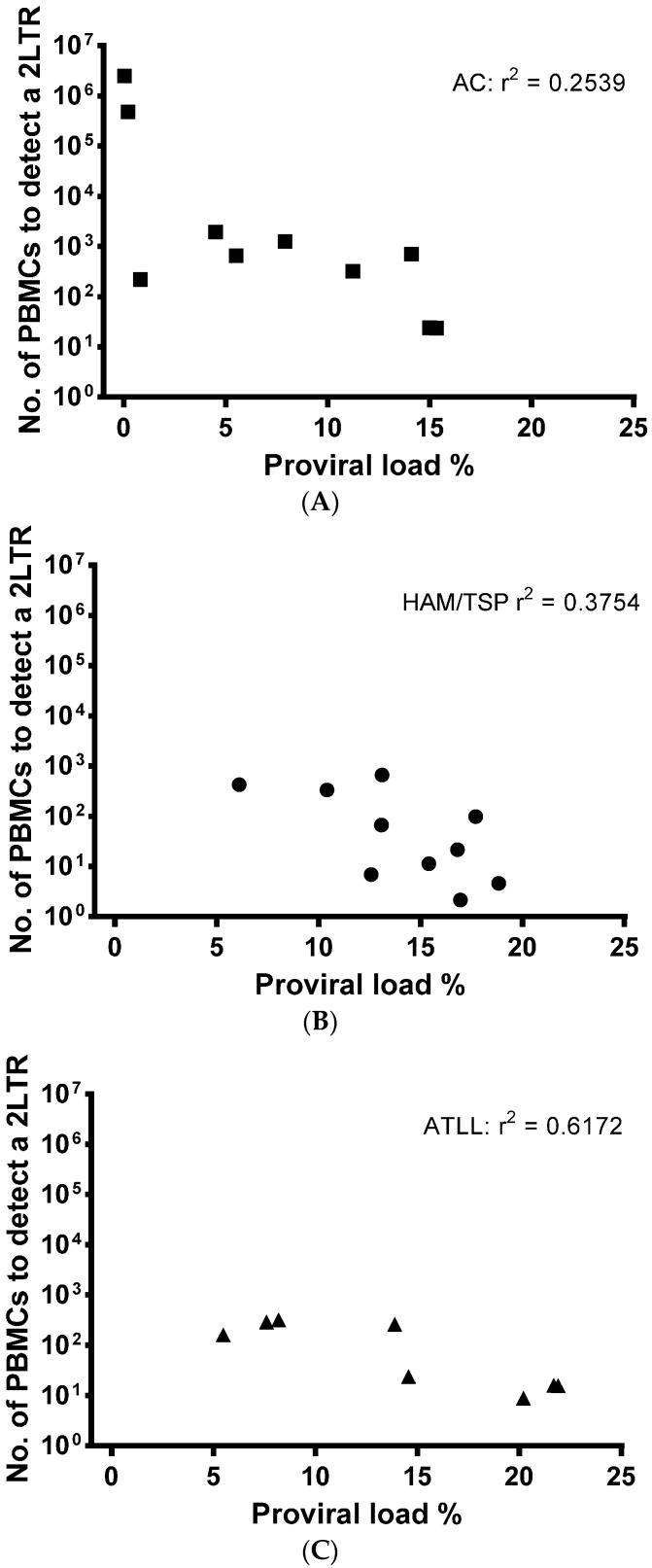
There is no correlation between PVL and 2LTR DNA circle levels. The number of PBMCs from patients with HTLV-1 required to detect 2LTR DNA circles in those diagnosed as asymptomatic (*n* = 10) (**A**); HAM/TSP (*n* = 10) (**B**) or ATLL (*n* = 8); (**C**) is plotted against the patients’ PVL (%). Correlation regression was calculated using line of best fit and r^2^ in PRISM software.

A correlation between PVL and 2LTR DNA circle concentration was not detected (Figure 3A–C). This indicates the on-going usage of viral RT and viral DNA production, despite established, chronic cellular infection, which is independent of the number of PBMCs infected.

### 3.4. Biomarker for HTLV Disease Progression

Stored DNA of PBMCs of one HTLV-1 positive patient with documented onset of definite HAM/TSP was tested. DNA samples were available from two time points: in 2004 when the individual was asymptomatic and in 2012 following the onset of HAM/TSP. The 2LTR DNA circle concentration was 2.06 fold higher at onset of disease, after adjusting for the rise in HTLV-1 PVL, which had increased 1.86 fold (1.62% to 3.01%) when symptomatic compared to the asymptomatic status (Figure S2). 1LTR DNA circles were undetectable in the DNA from this patient using classical or nested PCR.

## 4. Discussion

LTR DNA circles are used to detect on-going viral replication in HIV infected individuals receiving antiretroviral therapy. Two randomised clinical trials and two cohort studies of antiretroviral therapy in patients with HTLV-1 did not show a significant change in *in vivo* PVL [32,33,34,35], which is thought to be mainly maintained through clonal expansion. Typically, *in vivo* HTLV-1 DNA levels are established early in infection and do not vary over time [51]. However, very little is known about HTLV-1 RT usage, the production of *de novo* virus and LTR DNA circle by-products, its effects on the total HTLV-1 viral load, disease development and progression during chronic HTLV-1 infection.

HTLV-1 extrachromosomal DNA was reported to be present up to 24 months after virus integration in HTLV-1 positive HL-60 cell lines; the amount of extrachromosomal DNA fluctuated over time possibly due to the replication and re-infection capacity of the virus [52]. Clones with multiple integrated HTLV-1 DNA were those with persistent extrachromosomal DNA. This was presumed to be the result of intracellular virus production and cell re-infection [14,15,17,18]. Another group detected 2LTR DNA circles in HTLV-1 and -2 positive cell lines but only in the PBMCs of one of seven patients with HTLV-1 ATLL, and not in any asymptomatics or HAM/TSP patients [53]. We recently contributed to findings assessing 2LTR levels in organ transplant recipients from an HTLV-1 infected donor: 2LTR DNA circles were detectable in all three recipients and the levels largely mirrored PVL fluctuations [28] suggestive of acute infectious spread.

Twenty years on, the relative contribution of cell proliferation and infectious spread to HTLV-1 viral load in chronic infection remains relatively unknown. We therefore decided to explore a marker of the HTLV-1 viral replication activity, and detected 1 and 2LTR DNA circles in both cell lines and patients’ samples. Typically, 2LTR DNA circles are investigated in HIV-1 research and we confirm that 2LTR DNA circles were more consistently found in HTLV-1.

In order to increase specificity we aimed to capture mainly small molecule DNA through a selective purification methodology and by using a low concentration of DNA molecules (~ 400 ng). This reduced the risk of hybridization of any linear compounds and linear amplification, as suggested previously [54]. Traditionally the whole LTR DNA circle is not amplified due to its large product size (~ 10 kb), but the orientation of the specifically designed primers allows the amplification across one LTR (1LTR) or the junction of two LTRs (2LTR), which are flanked by viral genome only. This excludes the amplification of integrated DNA.

With the assumption that RT usage precedes LTR circle production, we attempted to inhibit entry and reverse transcription*.* In cell lines, LTR circle concentrations decreased in the presence of the most potent *ex vivo* RT inhibitor [30], TDF, but not with an HTLV-1 entry inhibitor P^cr^-400. This indicates recycling of viral RNA through persistent RT usage and LTR circle production in chronic infection, which is independent from new viral entry. Other publications also support the perception that HTLV-1 has a replicative potential *in vivo* via dendritic cell infection and potential continuous spread to lymphocytes [24,55,56].

Unlike previous reports, in our study LTR circles were detected not only in chronically infected cells lines, but also in the PBMCs of almost all HTLV-1 positive patients (46/47). The formation of LTR circles seems to be less common in HTLV-1 than in HIV-1 infected cells, which supports the perception that HTLV-1 is less replication active. This might provide the virus with survival benefits. It has been suggested that an accumulation of DNA circles is highly cytopathic, increasing cell death in HIV infected cells [14,57]. Also T cells containing less replicative viruses may more readily escape a HTLV-1 specific cytotoxic T cell response [58].

Although 1 and 2LTR DNA circles could be found in HTLV-1 infected patients, 2LTR circles were more commonly detected. The finding that 2LTR DNA circle concentration consistently differentiated symptomatic patients from ACs, independent of total, linear, DNA concentration, was surprising. This difference persisted, even when 2LTR DNA circle levels of ACs with high PVL were compared to patients with HAM/TSP or with chronic/cutaneous ATLL. 2LTR DNA circles did not differentiate between HAM/TSP and ATLL patients. In addition, we observed a significantly higher 2LTR DNA circle level in the patient with recent onset of HAM/TSP compared to their asymptomatic state. Together, these observations suggest an association between 2LTR DNA levels and clinical disease activity. To confirm this LTR DNA circles should be monitored longitudinally in a considerable cohort of ACs to measure variations of LTRs over time with and without disease onset.

These results not only indicate that HTLV-1 actively replicates in chronic infection, but also raise the following four questions. (1) How much does on-going RT usage contribute to PVL and promote cell-to-cell perhaps even human-to-human transmission? The reduction of LTR DNA circles with the RT inhibitor TDF *in vitro* poses the question (2) as to whether HTLV-1’s infectivity may be modifiable? (3) Could active viral replication contribute to pathogenesis and is viral replication increased in patients with active disease? Increased viral activity may lead to increased viral antigen expression and local recruitment of HTLV-1 specific cytotoxic T lymphocytes, cytokine release and tissue damage. 2LTR DNA circle levels may be a potential biomarker of disease onset/risk or treatment response; (4) Are HTLV-1 LTR DNA circles functional in themselves? It has been suggested that HIV-1 circles are transcriptionally active, producing functionally active proteins such as tat, env, nef [59,60,61,62,63,64].

## 5. Conclusions

Despite presumed latency or inactivity of HTLV-1, 1 and 2LTR DNA circles are present in chronically infected cell lines and in patient PBMCs, indicating ongoing viral RT usage. Monitoring 2LTR DNA circle concentration might be useful to identify early patients in the process of disease development and in this manuscript we provide novel methodology for this assessment. The clinical application of HTLV-1 2LTR DNA circles needs to be tested further in larger patient cohorts longitudinally and is likely to be achieved in multicentre collaborations.

## Figures and Tables

**Table 1:** Sequences of the primers and probes used.

Primer/Probe	Sequence	Product Size
1LTR probe	CTCTCACACGGCCTCATACAGTACTCTTCCTTTCATAGTTTACATCTCCTGTTTGAATTATTCCCTAGGCAATGGGCCAAATCTTTTCCCGTAGCGCTAG	100 bp
1LTR FWD	CTCTCACACGGCCTCATACA	940 bp
1LTR REV	CTAGCGCTACGGGAAAAGAT	
1LTR nested probe	CACCAACATCCCCATTTCTCGTACTCTTCCTTTCATAGTTTACATCTCCTGTTTGAATTATTCCCTAGGCAATGGGCCAAACTGGCAATGGGCCAAATCT	100 bp
1LTR nested FWD	CACCAACATCCCCATTTCTC	840 bp
1LTR nested REV	AGATTTGGCCCATTGC	
2LTR probe	ATGAGCCCCAAATATCCCCCGGGGGTACTCTTCCTTTCATAGTTTACATCTCCTGTTAGTTGAATTATTCCCTAGGCAGTTCTGCGCCGTTACAGATCGA	100 bp
2LTR FWD	ATGAGCCCCAAATATCCCCCGGGG	611 bp
2LTR REV	TCGATCTGTAACGGCGCAGAAC	
2LTR nested probe	AGCCACCGGGAACCACCCATGTACTCTTCCTTTCATAGTTTACATTTGTTTGAATTATTCCCTAGGCAATGGGCCAGGTCGAGACCGGGCCTTTGTC	100 bp
2LTR nested FWD	AGCCACCGGGAACCACCCAT	307 bp
2LTR nested REV	GACAAAGGCCCGGTCTCGACCT	

**Table 2:** Quantification and ratio calculation of 1 long terminal repeat (LTR) and 2LTR DNA circles in two cell lines carrying human T-lymphotropic virus type-1 (HTLV-1).

Cell Line	Limit of Detection in 1 μg DNA		Ratio of 1:2 LTR
	1LTR	2LTR	
MT-2	8.79 copies	14.67 copies	1:1.7
HuT 102	5.51 copies	11.03 copies	1:2

**Table 3:** 1LTR DNA circle detection in samples from patients with HTLV-1. Patients are denoted a code. VL% = HTLV-1 DNA copies/100 PBMCs, 1LTR% = number of 1LTR DNA circles/100 PBMCs. Code prefix H = ACs, prefix T = HAM/TSP, prefix L = ATLL. * denotes acute ATLL.

Patient	VL% (Range)	1LTR	1LTR%
HBK	129	+	0
HBU	3.94	+	0
HCT	0.08	+	0.0023
HEF	2.39	+	0
HT	23.4	-	0
HX	0.02	-	0
**ACs mean**	**26.5 (0.02–129)**		
TAF	76.8	+	0.0048
TAN	16	+	0.0048
TAQ	12.3	+	0.0023
TAS	17.4	+	0.00048
TBW	12.8	+	0.0023
TBX	13.28	+	0.000048
TBR	22.9	+	0
TBJ	51	-	0
TBU	10.2	-	0
TCD	43.7	-	0
**HAM mean**	**27.6 (10–77)**		
LER	76.75	+	0.00023
LEV*	124.7	+	0.11547
LEY	77.1	+	0.0048
LP4	96.3	-	0
**ATLL mean**	**94 (77–125)**		

**Table 4:** 2LTR DNA circle detection in samples from patients with HTLV-1. Patients are denoted a code. VL% = HTLV-1 DNA copies/100 PBMCs, 2LTR% = number of 2LTR DNA circles/100 PBMCs. Code prefix H = ACs, prefix T = HAM/TSP, prefix L = ATLL. * denotes acute ATLL.

Patient	VL% (Range)	2LTR	2LTR%
HBU	4.5	+	0.0005184
HBZ	5.5	+	0.0015246
HBF	7.9	+	0.00079
HBE	14.1	+	0.00141
HAO	0.21	+	0.0000021
HBK	10.3	Nested +	
HBX	11.23	+	0.003112956
HCH	15	+	0.04158
HDH	0.04	+	0.0000004
HDO	0.01	-	Undetectable
HDY	0.06	Nested +	
HFE	15.33	+	0.04249476
HT-UV1	0.82	+	0.00454608
**ACs mean**	**6.54 (0.01–15)**		
TBP	6.1	+	0.0023424
TBW	13.1	+	0.00150912
TBG	16.8	+	0.0465696
TBA	17.7	+	0.0101952
TAL	15.4	+	0.088704
TAT	10.4	+	0.0029952
TBZ	18.83	+	0.2169216
TCJ	16.95	+	0.469854
TCP	13.07	+	0.01505664
TCQ	12.57	+	0.1448064
**HAM mean**	**14.09 (6–19)**		
LEU	7.6	+	0.00347776
LGH	13.88	+	0.003847536
LGA	21.68	+	0.0624384
LFE	15.2	+	0.210672
TBX	14.57	+	0.00839232
LFK *	8.2	+	0.0031488
LFA *	21.9	+	0.063072
LFP *	5.48	+	0.00631296
**ATLL mean**	**13.56 (5–22)**		

## Supplementary Materials

The following are available online at http://www.mdpi.com/1999-4915/8/3/80/s1. Table S1: Demographics of the study participants; Figure S1: Structures of extrachromosomal DNA in HTLV infected cells and primer design rational; Figure S2: PVL and 2LTR levels increase in an incident patient progressing from asymptomatic HTLV-1 carrier to being diagnosed with HAM.
