# Supplementary material for: Long Terminal Repeat Circular DNA as Markers of Active Viral Replication of Human T Lymphotropic Virus-1 in Vivo
Source: Viruses. 2016 Mar 14;8(3):80. doi: 10.3390/v8030080 (PMC4810270; doi:10.3390/v8030080)
Supplement: Supplementary file 1 [file viruses-08-00080-s001.pdf]

Supplementary Materials: Long Terminal Repeat Circular DNA as Markers of Active Viral Replication of Human T Lymphotropic Virus-1 *in Vivo*

James M Fox, Silva Hilburn, Maria-Antonietta Demontis, David W Brighty, Maria Fernanda Rios Grassi, Bernardo Galvão-Castro, Graham P Taylor and Fabiola Martin

Table S1. Demographics of the patients with HTLV-1 from whom DNA samples for LTR quantification were taken.

Demographics		Frequency (%)	Range
	Female	33 (70)	
	Afro-Caribbean	37 (80)	
	African	4 (8%)	
	Caucasian	4 (8 %)	
	Persian	1 (2%)	
	South American	1 (2%)	
Age		Years	
	Average age at sampling	56.1	19–80

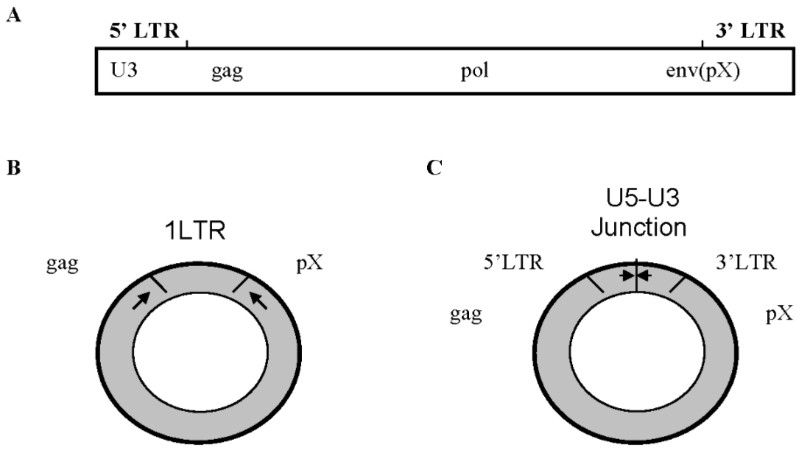

Figure S1. Structures of extrachromosomal DNA in HTLV infected cells and primer design rational. Linear viral DNA (**A**) is synthesized in the cytoplasm after cell infection and can circularise to form 1- (**B**) or 2-LTR DNA circles (**C**). 1LTR DNA circles are formed through the deletion of either LTR; 2LTR DNA circles are formed at the U3-U5 junction where the LTRs from each end of the HTLV-1 DNA connect. Specific primer pairs (arrows) were designed to selectively amplify circularised DNA (sequences are shown in Table 1). LTR: long terminal repeats; gag, pol, env(pX): gene regions coding for viral proteins.

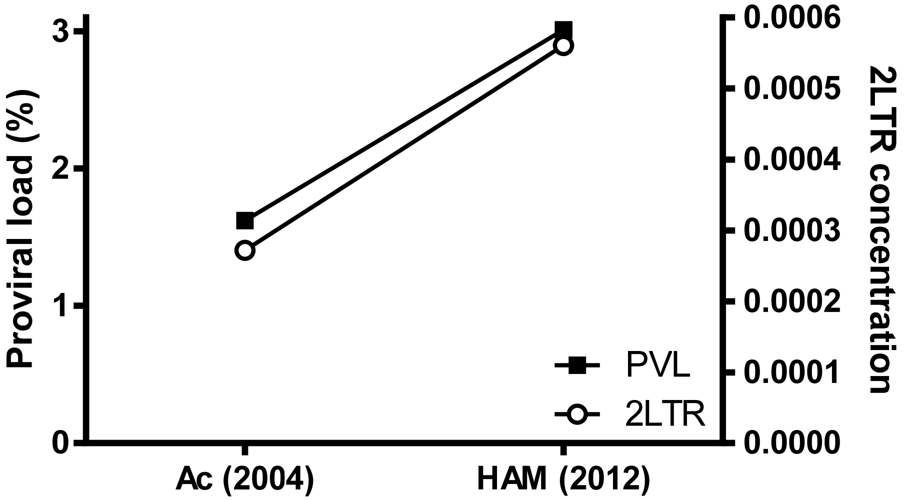

Figure S2. PVL and 2LTR DNA circle levels increase in an incident patient progressing from asymptomatic HTLV-1 carrier to being diagnosed with HAM. The PVL (■) and 2LTR levels (○) were quantified in PBMCs from an incident asymptomatic patient in 2004 and from the same patient after disease progression and HAM diagnosis in 2012. Incident patient demographics: male; 20 years of age at diagnosis; black ethnicity (skin colour, self-definition). His risk factors were: breast-feeding from an HTLV positive mother and his brother was also HTLV positive. Blood transfusion denied. His date of diagnosis was on his enrolment into the cohort: 10 October 2004 when he was asymptomatic. The patient was seen again on 9 May 2009 and he was still asymptomatic but Rossolimo's sign was present. Onset of symptoms: 9 September 2011 (HAM/TSP).

© 2016 by the authors; licensee MDPI, Basel, Switzerland. This article is an open access article distributed under the terms and conditions of the Creative Commons by Attribution (CC-BY) license (http://creativecommons.org/licenses/by/4.0/).
